# Dietary Intake of n-3 Polyunsaturated Fatty Acids Prior to a Mild Traumatic Brain Injury Demonstrates a Dose–Response Effect for Neuroprotective Benefits in Male C57BL/6 Mice

**DOI:** 10.1016/j.cdnut.2025.107476

**Published:** 2025-05-27

**Authors:** Cody AC Lust, Jessi Lau, Lyn M Hillyer, Margo Mountjoy, Lindsay A Robinson, David WL Ma

**Affiliations:** 1 Department of Human Health and Nutritional Sciences, University of Guelph, Guelph, ON, Canada; 2 Department of Family Medicine, McMaster University, Hamilton, ON, Canada

**Keywords:** omega-3, omega-6, concussion, neuroprotective, mice, diet, dose–response

## Abstract

**Background:**

Of the ∼40 million cases of mild traumatic brain injury (mTBI) documented globally each year, the majority are sustained during sports and recreational activities. Evidence has suggested that the use of nutritional supplementation, notably n-3 polyunsaturated fatty acids (PUFA), can provide neuroprotective benefits.

**Objectives:**

This study sought to examine the dose–response of consuming an n-3 PUFA diet prior to an mTBI on recovery and functional performance measures in mice.

**Methods:**

For 5 wk, male C57BL6/J mice were fed ad libitum on isocaloric diets high in n-3 PUFA (3N3), moderate n-3 PUFA (1N3), or n-6 PUFA (0N3) as a control. At 9–10 wk of age, mice were anesthetized in an induction chamber prior to receiving a mild brain injury induced using a weight-drop injury model. A one-way analysis of variance (ANOVA) with Tukey’s post hoc analysis was used to assess between-group differences in functional recovery measures of righting reflex and time to seek) immediately after injury. A repeated measures ANOVA was conducted to determine the effect of diet groups and time on functional performance measures indicated by neurological severity score (NSS) 1, 4, 24, 48, 72, and 168 h (H) post-mTBI.

**Results:**

There were no significant differences in recovery measures; however, the 3N3 group demonstrated the fastest recovery post-mTBI and had significantly improved functional performance (*P <* 0.05) compared with the 0N3 group determined by NSS testing. A one-way ANOVA with Tukey’s post hoc indicated that the 3N3 group had significantly improved functional performance (*P <* 0.05) at 4H post-mTBI compared with the 0N3 group.

**Conclusions:**

Overall, these results show that dietary n-3 PUFA confer neuroprotective benefits in mice resulting in significantly improved functional outcomes. This work is relevant to clinical practitioners, athletes, and the general population who aim to support their neurological health through dietary or supplementary n-3 PUFA.

## Introduction

There are over 40 million cases of traumatic brain injury (TBI) documented globally each year with the mild form of TBI (mTBI) accounting for >80% of all reports [1,2]. The lifetime burden of healthcare costs and losses in productivity because of all forms of TBI already extends into the tens of billions of dollars in North America, making effective strategies to mitigate brain injuries an important health and economic concern [3,4]. Historically, researchers and practitioners have used the terms mTBI and concussion interchangeably when discussing mild brain injuries, particularly in a sports-related context [5]. Although both concussions and mTBI are on the mild end of the brain injury spectrum, The American Congress of Rehabilitation Medicine considers a concussion to be a milder form of TBI that lacks macrostructural injury indicated on neuroimaging scans [6]. Both concussions and mTBI are commonly induced by a direct/indirect biomechanical force applied to the head or neck that results in both physical trauma and increased neuroinflammation [7]. Even if macrostructural damage is not present on neuroimaging scans, microstructural damage typically occurs as a result of the primary injury to the brain leading to disruptions of neuronal cell membranes and the blood-brain barrier (BBB), axonal shearing, and microbleeding [8,9]. The resulting damage and prolonged neuroinflammation, known as the secondary injury, can cause acute and even chronic disruptions to cerebral homeostasis leading to impaired cognitive, emotional, and physical health after an mTBI and concussion [10,11]. Thus, preventative interventions that mitigate the biochemical and cellular events that are triggered after a brain injury can be a unique avenue for neuroprotection.

Brain injuries typically occur because of unexpected events such as falls, motor vehicle accidents, and sport-related injuries, limiting many practical options for preventative intervention [12]. Emerging evidence has suggested that the use of nutritional supplementation can provide neuroprotective benefits by priming the cellular microenvironment of the brain prior to injury [13,14]. The long-chain n-3 polyunsaturated fatty acids (PUFA) eicosapentaenoic acid (EPA) and docosahexaneoic (DHA) have demonstrated anti-inflammatory effects in the brain [15,16]. DHA is an essential component of neural development and healthy brain functioning, making up >95% of the total n-3 PUFA content in the brain, with increased dietary intakes correlating with improved cognitive functioning throughout life [17]. High concentrations of DHA are found in phospholipid membranes, allowing DHA to modulate the BBB and act as a mediator of several inflammatory pathways within the brain and central nervous system [15,18]. There is a growing body of literature demonstrating that n-3 PUFA supplementation can improve functional and behavioral outcomes by reducing neuroinflammation when taken after a brain injury [19,20]. A systematic review by Patch et al. [21] highlights a positive dose–response effect of n-3 PUFA supplementation when given post-mTBI that correlates with improved cognitive performance and reduced markers of inflammation in rodents. Comparatively, there is less research on the effects of n-3 PUFA when intake is prior to a brain injury. Increasing n-3 PUFA intake in the general population and athletes alike has been found to reduce the risk of cardiovascular disease and provide neuroprotective benefits [22,23]. Our laboratory has previously reported that *fat-1* mice, which endogenously convert n-6 PUFA from the diet to n-3 PUFA, have improved functional outcomes when brain levels of n-3 PUFA are increased prior to receiving a brain injury [24]. However, humans are unable to convert n-3 PUFA endogenously, and we must obtain them through our diet or supplementation [25]. Despite accessibility of foods and supplements containing n-3 PUFA, intake and serum levels of EPA and DHA in North America are low, illuminating a nutritional gap that can be readily addressed [26]. Thus, our study sought to examine the neuroprotective efficacy of consuming a diet high in n-3 PUFA prior to the induction of an mTBI using a weight-drop injury model. Male C57BL/6 mice were fed ad libitum 1 of 3 isocaloric diets containing 2 different concentrations of n-3 PUFA or a control diet high in n-6 PUFA, without n-3 PUFA, for 5 wk prior to receiving an mTBI. On the basis of our previous findings [24], we hypothesize a positive dose–response effect of increasing n-3 PUFA in the diet, resulting in improved functional outcomes and reduced markers of cerebral microinjury post-mTBI.

## Methods

### Animals

All procedures pertaining to the handling, care, and experimental protocols related to this project were approved by the Institutional Animal Care Committee at the University of Guelph (AUP #4207), which is accredited by the Canadian Council on Animal Care. All male C57BL/6 mice used in this study were received from Charles River Laboratories at 4–5 wk of age. Mice were fed ad libitum a diet consisting of either 10% w/w safflower oil (0N3) containing n-6 PUFA, 1% w/w menhaden [moderate n-3 PUFA (1N3)] oil, or 3% w/w menhaden [high n-3 PUFA (3N3)] oil (Research Diets) for a minimum of 5-wk pre-mTBI and 1-wk post-mTBI until termination. The fatty acid composition of the 3 diets is described in Table 1. Sham mice were separated into the same 3 diet groups and were also anesthetized in the same manner but were not subjected to a weight-drop injury. All experimental groups (receiving an mTBI) and sham groups had a sample size of *n =* 8.

**TABLE 1:** Mean fatty acid composition of diets.

Fatty acid	3% Menhaden oil	1% Menhaden oil	10% Safflower oil
12:0	0.00	0.00	0.00
14:0	2.88	1.37	0.00
15:0	0.00	0.00	0.00
16:0	9.63	7.84	6.74
16:1c9	4.09	1.77	0.00
17:1c10	0.00	0.00	0.00
18:0	3.12	3.06	3.08
18:1c9	12.25	13.91	14.91
18:1c11	1.88	1.58	1.19
18:2n6	49.20	64.18	71.76
18:3n6	0.00	0.00	0.00
19:0	0.00	0.00	0.00
19:1c7	0.00	0.00	0.00
18:3n3	0.69	0.00	0.00
18:4n3	1.22	0.55	0.00
20:0	0.22	0.00	1.11
20:1c5&8	0.00	0.00	0.00
20:1c11	0.90	0.00	0.00
20:2n6	0.00	0.00	0.00
20:3n6	0.00	0.00	0.00
20:4n6	0.15	0.00	0.00
20:3n3	0.00	0.00	0.00
20:5n3	4.24	1.80	0.00
22:0	0.00	0.00	0.20
22:1n9	0.00	0.00	0.00
22:2n6	0.00	0.00	0.00
22:4n6	0.00	0.00	0.00
22:5n3	3.09	0.00	0.00
24:0	0.00	0.00	0.00
22:6n3	4.70	2.90	0.00
24:1n9	0.00	0.00	0.00
Total n-6	49.35	64.18	71.76
Total n-3	13.93	5.24	0.00

Fatty acid composition (%) of safflower and menhaden oil diets. Three separate pellets per diet were analyzed and quantified by gas chromatography.

## Inducing the mTBI

On the basis of the methodology of Flierl et al. [27] and Kane et al. [28], once mice reached ∼9 wk of age, they were exposed to a singular weight-drop injury, resulting in an mTBI. All mice were first placed in an anesthetic chamber and induced via the administration of 4% Baxter isoflurane (Cat#19476, CMDV) vaporized in oxygen. After 60–90 s of anesthetic administration and the surgical plane was reached, mice were removed from the anesthetic chamber and placed on a nose cone, where isoflurane concentration was reduced from 4% to a maintenance level of 1.5%. Once each mouse reached a total of 4 min of exposure to isoflurane, the anesthetic was stopped and the mice were then immediately placed under the weight-drop apparatus. The weight-drop apparatus consisted of a 100 g steel weight (1.3 cm in diameter) attached via nylon fly fishing line that was released from a height of 163 cm and guided vertically through a PVC tube (20 mm × 163 cm). A single concussive impact was achieved by placing the anesthetized mouse chest-down below the weight-drop apparatus on a section of aluminum foil, with a slit on each side to ensure collapse. The platform and mouse were positioned ∼10 cm above foam padding, which was enclosed in a Plexiglass housing to mitigate additional injury upon landing. A diagram demonstrating an example of the weight-drop apparatus can be found in Kane et al. [28]. The alignment of the mouse on the collapsible platform directly below the PVC tubing ensured that the weight fell directly onto the dorsal surface of the cranium, approximately between the bregma and lambda, delivering a force of impact clinically relevant to closed cranium scenarios [29].

## Time to Movement: Righting Reflex and Time to Seek

After the head impact, each mouse was immediately moved to an isolated containment enclosure and positioned flat on their dorsal side/back. The righting reflex (RR) duration was assessed for each mouse, which measured the time required to reorient itself, transitioning from dorsal to ventral positioning. In addition to the RR, time to seek (TTS) was evaluated as the time from impact to when mice exhibited a voluntary exploring/seeking behavior within the isolated containment enclosure. Akin to the RR parameter assessed by Kane et al. [28], the TTS measure was recorded to evaluate the voluntary movement of the mice around their enclosure, which served as an indicator of neurological recovery.

## Neurological Severity Score

As previously described in Flierl et al. [27], the recovery and functional capability of mice post-mTBI was scored using a validated and comprehensive assessment known as the neurological severity score (NSS) test. The NSS test encompasses 10 distinct clinical tasks that assess motor, cognitive, and neurobehavioral performance in mice after a brain injury. On the basis of the established criteria described in Table 2 [27], a researcher would evaluate mice using a 0 (pass) or 1 (fail) scoring system on whether a mouse was able/unable to perform a task; a score of 0 denotes unimpaired neurological function and a score of 10 denotes maximal neurological impairment. Six NSS assessments were carried out on all mice at 1, 4, 24, 48, 72, and 168 hours (H) post-mTBI, which on completion were terminated for tissue collection.

**TABLE 2:** Description of the tasks comprising the neurological severity score.

Description of task	Score
Exit circle (max 3 min)	0 | 1
Straight walk	0 | 1
Seeking behavior	0 | 1
Startle reflex	0 | 1
Monoparesis/hemiparesis	0 | 1
Beam balance (min. 10 s)	0 | 1
Beam walk: 3 cm width (max 3 min)	0 | 1
Beam walk: 2 cm width (max 3 min)	0 | 1
Beam walk: 1 cm width (max 3 min)	0 | 1
Round stick balance (min. 10 s)	0 | 1
Total	/10

As described in Flierl et al. [27] and scored on a pass-fail system (0 = pass, 1 = fail) determined by observation from the examiner.

## Tissue Collection

Mice were killed via CO_2_ asphyxiation at 168H post-mTBI and immediately on termination the entirety of the brain was collected. The whole brain was fixed in formaldehyde and stored at 4 °C immediately after collection.

## Immunohistochemical Staining

Whole brains from each of the 3 experimental groups (*n =* 6), with a matching sham, were fixed in 4% formaldehyde and processed in a tissue processor overnight for immunohistochemical (IHC) staining. Brain tissue was embedded in a paraffin block from which 5 μm sections were sliced along the dorsoventral plane (Leica RM2235 microtome) and the free-floating tissue sections were then fixed onto Superfrost Plus slides (Fisher, Cat#12-550-15). Two slides were allocated to evaluate each of the following measures: *1*) Prussian Blue (PB) stain to evaluate cerebral microbleeds, and *2*) the presence of glial fibrillary acidic protein (GFAP) using a ready-to-use kit. PB staining was conducted on sections 55–60 μm below the site of impact (most dorsal portion of the brain) and GFAP staining was conducted on sections 65–70 μm below the site of impact. Slides that were designated for PB staining were deparaffinized and hydrated using distilled water before submersion in a solution comprising equal parts of 20% HCl (Fisher, Cat #A144-500) and 10% potassium hexacyanoferrate (II) trihydrate (Sigma-Aldrich Canada Co., CAN, Cat #P3289-5G) for 20 min. Slides were subsequently rinsed in 2 successive changes of water and counterstained with nuclear fast red (Ricca Chemical Company, Cat #R5463200-500A) for a duration of 5 min, followed by rinsing in 3 successive changes of water. Finally, the presence of GFAP was evaluated using the ProteinTech IHCeasy GFAP Ready-To-Use IHC Kit (Cat #KHC0002). The protocol for the staining of the designated slides followed the protocol provided by ProteinTech.

After processing for each respective stain of PB and GFAP all slides underwent a sequence of baths in the following order: dehydration in 95% isopropanol (Fisher HistoPrep, Cat #HC5001GAL); 2 rounds in isopropanol for dehydration; and 3 rounds of xylene (Fisher HistoPrep, Cat #HC7001GAL), each for a duration of 120 s. On completion, the sections were mounted using Cytoseal XYL (Epredia, Cat #8312-4) and cover slipped (Fisher, Cat #12-541-023CA) and left to dry prior to viewing. Two independent observers evaluated all slides separately to determine PB clusters and GFAP appearance using the same Nikon Eclipse TS100 microscope at 20× and 40× original magnification, respectively.

## Data Analysis

All statistical analyses were conducted in SAS OnDemand for Academics (SAS Institute Inc.). A one-way analysis of variance (ANOVA) with Tukey’s post hoc analysis was used to assess between-group differences in RR and TTS measures. A repeated measures ANOVA was conducted to determine the effects of the 3 diet groups and time on NSS measures post-mTBI. A significant effect (*P* < 0.05) was found in the model, between groups, and time (*P <* 0.05) following the repeated measures ANOVA. A subsequent one-way ANOVA was conducted at each timepoint (1H, 4H, 24H, 48H, 72H, and 168H) with Tukey’s post hoc analysis to determine differences between diet groups.

The trauma induced in our brain injury model was mild resulting in limited expression of cerebral microinjury following IHC staining. Thus, positive samples which indicated microinjury were qualitatively similar and differences were unable to be statistically analyzed.

## Results

### RR and Time to Seek

**FIGURE 1:**
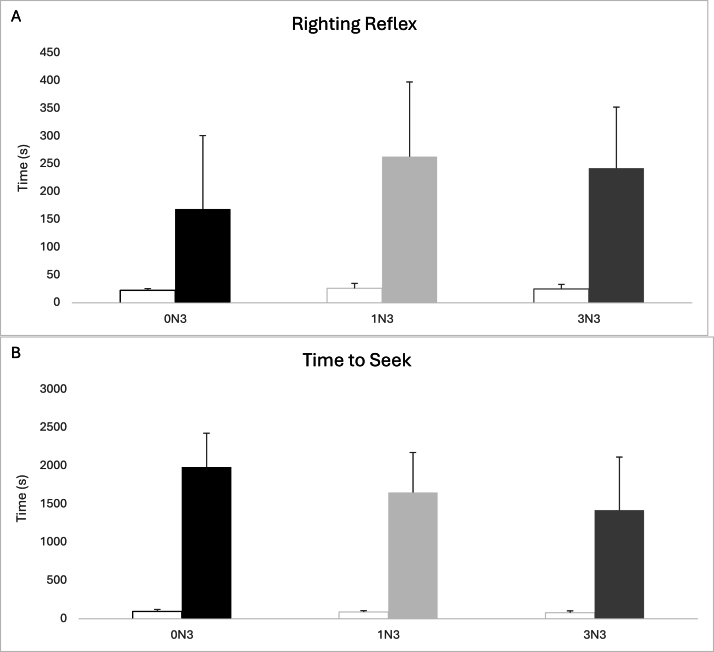
Functional recovery times post-mTBI across diet groups. All groups were significantly greater (*P <* 0.05) than sham mice (left bar), which were excluded from statistical analysis. (A) No significant effects were found for righting reflex between groups. (B) No significant effects were found for time to seek between groups. Error bars represent SD. *n* = 8 for all groups. 0N3, n-6 PUFA diet; 1N3, moderate n-3 PUFA diet; 3N3, high n-3 PUFA diet; mTBI, mild traumatic brain injury.

As expected, all experimental groups had significantly higher measured times (*P <* 0.05) for RR and TTS compared with sham mice (not displayed) in both Figure 1A and B. A one-way ANOVA with Tukey’s post hoc analysis found no significant effect of diet (*P <* 0.05) for either RR or TTS measures.

**FIGURE 2:**
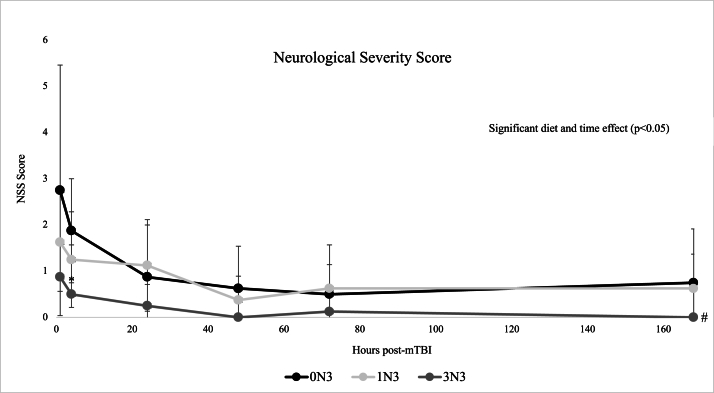
Mean NSS measures between groups over a 168H period. All sham mice scored 0 (lowest possible score) and were not included in the statistical analysis. NSS was conducted 1, 4, 24, 48, 72, and 168H post-mTBI. A repeated measures ANOVA found a statistically significant model, group, and time effect (*P <* 0.05). Error bars represent SD. *n* = 8 for all groups. ^#^ Repeated measures ANOVA found the 3N3 group to be significantly lower (*P <* 0.05) than 0N3. ∗A one-way ANOVA was conducted at each timepoint and found 3N3 to be significantly lower (*P <* 0.05) than 0N3 at 4H. 0N3, n-6 PUFA diet; 1N3, moderate n-3 PUFA diet; 3N3, high n-3 PUFA diet; ANOVA, analysis of variance; H, hours; mTBI, mild traumatic brain injury; NSS, neurological severity score.

A repeated measures ANOVA found a statistically significant model, diet, and time effect (*P <* 0.05) for NSS measures between diet groups in Figure 2. NSS measures of mice consuming the 3N3 diet were significantly lower (*P <* 0.05) than those of mice consuming the 0N3 diet. As no significant interaction effect was found, a one-way ANOVA was conducted for each timepoint and at 4H post-mTBI, mice fed the 3N3 diet scored significantly lower (*P <* 0.05) on the NSS than the 0N3 diet, indicating improved neurological function. Over the course of the 7-d post-mTBI, the n-3 PUFA containing diet groups (3N3 and 1N3) consistently demonstrated lower mean NSS scores than mice consuming the 0N3 diet (10% w/w safflower oil) in Figure 2.

### IHC staining

**FIGURE 3:**
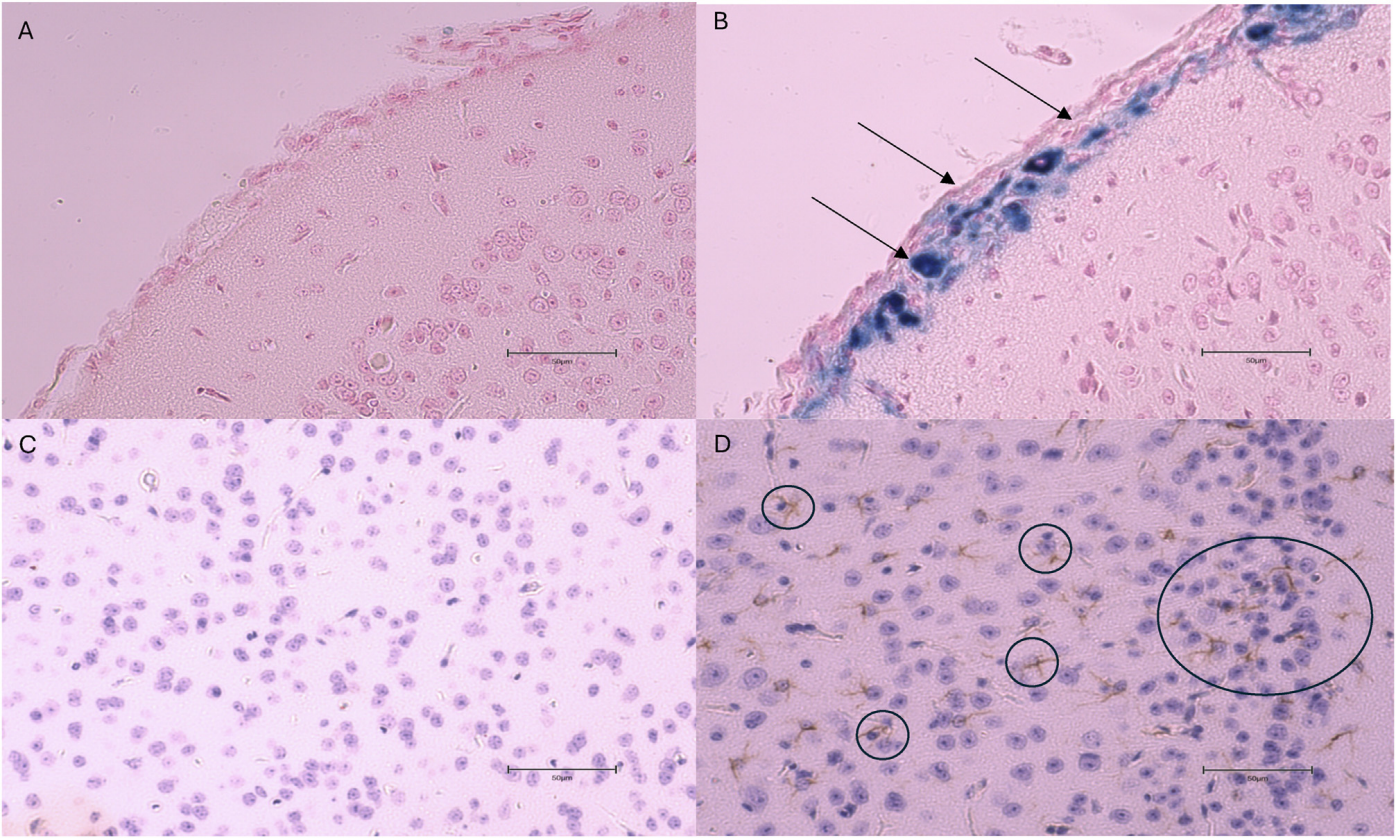
Immunohistochemical staining images. (A) is a representative image of Prussian Blue staining for a sham mouse. (B) A representative image of a positive Prussian Blue stain (indicated by the black arrows) after mTBI. (C) A representative image of glial fibrillary acidic protein staining for a sham mouse. (D) A representative image of a positive glial fibrillary acidic protein stain (indicated by the black circles) after mTBI. Images were taken using the EVOS M7000 Imaging System (Invitrogen #AMF7000) at 40× magnification. All sections were taken along the dorsoventral plane of the brain. Scale bars: 50 μm (A–D). mTBI, mild traumatic brain injury.

The severity of microinjury in the brain samples assessed was mild compared with other TBI models as there was limited staining of positive cells from samples in the present study [30]. Detectable staining of GFAP and PB was only found in ≤ 3 samples among the 6 samples assessed per group resulting in no statistical difference in the number of stained cells between groups. The 0N3 group had 3 GFAP and 2 PB-positive samples. The 1N3 group had only 1 GFAP and 0 PB-positive samples. The 3N3 group had 2 GFAP and 1 PB-positive sample. Images of IHC stains from sham mice and those that displayed positive expression of PB and GFAP are shown in Figure 3. Each experimental group had a total of *n =* 6.

## Discussion

This study demonstrates a positive and significant (*P <* 0.05) dose–response effect of dietary intake of n-3 PUFA prior to an mTBI confers neuroprotective benefits resulting in improved functional outcomes in male C57BL/6 mice. Our results add to the growing body of literature supporting the use of dietary n-3 PUFA as a safe, practical, and effective means for providing neuroprotective benefits prior to an mTBI.

Our findings are consistent with our previous work and others using brain injury models showing that n-3 PUFA diets consumed prior to a brain injury results in improved recovery following injury [24,31,32]. Recent studies in American-style football players have also found neuroprotective benefits in participants who received n-3 PUFA supplementation prior to the start and during activity [33,34]. The RR measure is a commonly used indicator of injury severity in mTBI models but has shown to be highly variable across different animal studies [35]. We modified the RR measure described in Kane et al. [28] to include a separate measure of TTS that reflects the restoration of both motor and cognitive functioning after an mTBI. There was no statistically significant difference found between groups for RR or TTS measures, but mice fed the 3N3 diet began to actively seek around their enclosure ∼10 min faster than mice fed the 0N3 diet post-mTBI (3N3: 1420.5 ± 442.4; 0N3: 1985.1 ± 695.1 s), as shown in Figure 1B. Although the result was not statistically significant, this demonstrates a potential functional improvement in acute neurological recovery of mice fed a high n-3 PUFA diet compared with a n-6 PUFA diet. The NSS testing method utilized in our study has been previously validated in animal models and provides a more comprehensive motor, cognitive, and behavioral outlook compared with the TTS measure and other functional tests such as the wire grip or rotarod [27]. A lower score on the NSS test has a strong correlation with reduced severity of structural damage to the brain, faster recovery, and improved neurological function post-mTBI [27,36]. Supporting the improved neurological recovery reflected by the TTS measure, there was a significantly lower (*P <* 0.05) NSS measure found in mice in the 3N3 group shown in Figure 2. The most pronounced differences between our diet groups were seen over the first 24H, where the 3N3 group had a lower (*P <* 0.05) NSS measure at 4H than the 0N3 group (3N3: 0.5 ± 1.1; 0N3: 1.9 ± 1.1). NSS measures in all diet groups began to plateau at 24H through to the last measure at 168H. After an mTBI, a 24H minimum of both physical and cognitive rest is recommended by specialists before engaging in any rehabilitation efforts or returning to school/the workplace [37]. The acute (≤24H) phase post-mTBI is thought to reflect the peak injury response where the greatest increase in neuroinflammation is found impacting both cognitive and functional outcomes [38,39]. The significantly lower NSS measures found in our n-3 PUFA diet groups within the first 24H could translate to an earlier return to work, school, or sport for individuals, minimizing productivity loss. This is particularly relevant to athletes playing contact sports, such as American-style football players described in Heileson et al. [33] and Raikes et al. [34] where rates of brain injuries are quite high compared with other sports. Further follow-up and validation studies are still required to better support this theory. Despite the increased risk of brain injury, even elite-level American-style football players have low serum levels of EPA and DHA that is also reflected in the general population at-large in North America [26,40]. Our laboratory has previously published findings that American-style football players can tolerate very high levels of DHA supplementation (6 g/d) and demonstrate a significant dose–response of serum DHA concentration over the course of a competitive season [41]. Russell et al. [42] reported that rats with low brain DHA content had poor sensorimotor outcomes after a TBI, reflecting the positive dose–response demonstrated with lower NSS measures in the 3N3 group compared with the 1N3 group in our study. Thus, we suggest that there is a positive dose–response effect of dietary n-3 PUFA to confer neuroprotective benefits, which acts as additional “nutritional armor” to protect the brain prior to the occurrence of an injury [43]. n-3 PUFA interact with multiple pathways implicated in the complex secondary injury sequelae of mTBI that can prime the cerebral microenvironment to reduce neuroinflammation and promote a pro-resolving injury response [[44], [45], [46]]. Tang et al. [47] suggest that the primary neuroprotective benefits of DHA are exerted through regulating the toll-like receptor 4 and NFκB signaling pathway to downregulate neuroinflammation. On the basis of the results reported herein, however, we are unable to specify the mechanism(s) by which n-3 PUFA are acting that, in turn, translates to the observed improvements in cognitive and functional outcomes. Additional studies assessing specific neural mechanisms influenced by n-3 PUFA are needed to fully understand their effects on mTBI pathology [14,48].

Evaluation of brain sections after IHC staining demonstrated minimal variation in the overall presence of GFAP or PB clusters in our study, and no statistical difference between groups was found. GFAP is released in response to astrocyte injury in the brain and is a common biomarker used for the diagnosis and prognosis of mTBIs [49]. PB staining identifies microbleeding present in the brain, which can indicate neuronal dysfunction and further complications within the cortex and subcortical structures [50]. Compared with other models of brain injury that also assessed the presence of GFAP and PB, the injury induced in our model was on the milder end of the TBI spectrum [30,51]. Mice that were fed a diet high in n-6 PUFA demonstrated a greater number of positive GFAP and PB samples than the 2 n-3 PUFA diets 168H post-mTBI. Previous studies reported that n-3 PUFA supplementation has been able to reduce the presence of GFAP in the brain after a TBI [19,47,52]. Previous results published from our laboratory group in Lecques et al. [24] found that *fat-1* mice had reduced PB clusters 168H post-mTBI, but this similarly did not demonstrate a statistically significant difference.

Although we do not report the concentration of n-3 PUFA in the brain of diet groups for this study, our results suggest that dietary intake of n-3 PUFA confers similar neuroprotective benefits as *fat-1* mice with increased brain DHA content. Previous research from our laboratory has shown that mice fed an n-3 PUFA-enriched diet for 5 wk demonstrated similar concentrations of DHA in the brain to that of their *fat-1* counterparts [53]. We posit that increased DHA content in the brain produced endogenously or via dietary intake modulates the same cellular mechanisms, resulting in the similar functional improvements of NSS measures demonstrated in both our studies. Due to the inability of humans to endogenously produce sufficient quantities of EPA and DHA, our current model of dietary n-3 PUFA consumption is a more accurate reflection of a feasible intervention to promote neuroprotective benefits in humans. The majority of dietary n-3 PUFA supplementation in the literature has taken place post brain injury and more evidence for the preventative benefits of n-3 PUFA intake prior to the occurrence of a brain injury is still needed. We recommend that future studies examining the neuroprotective efficacy of dietary n-3 PUFA also include additional peripheral biomarkers that have shown to correlate with diagnostic and prognostic outcomes in human models [54]. Such markers include serum levels of GFAP, neurofilament light, and the IL class of cytokines including IL-6, IL-1β, and IL-10 [54].

### Limitations

Our study has provided novel insights into the neuroprotective benefits of an n-3 PUFA diet, but it is not without limitations. Only male mice were used in this study and previous research has found that male and female mice exhibit differential levels of functional impairment post-TBI [51]. n-3 PUFA supplementation has also shown differential increases in serum levels of EPA and DHA between males and females, potentially altering the neuroprotective efficacy between sexes [55]. The 2 stains (PB and GFAP) used in this study could not be conducted on the same section of brain tissue; thus, a slight variance in the depth from the site of impact is noted. Finally, the half-life of GFAP in serum is reported to be 24–36H and our samples were assessed 168H post-mTBI [56]. The extended time from mTBI to IHC assessment coupled with the mild injury induced by our weight-drop mechanism could help explain why there was a low expression of GFAP. Including more acute measures (≤24H) of functional and biochemical outcomes could provide a more robust effect in future work.

In conclusion, the results from this study suggest that dietary intake of n-3 PUFA prior to an mTBI confers neuroprotective benefits resulting in significantly improved cognitive and functional outcomes in mice. Our results also suggest that n-3 PUFA can provide moderate benefits for protecting the structural integrity of the brain as expressed by reduced markers of microinjury; however, more research is needed to support this finding. We have added to the growing body of literature supporting the use of dietary n-3 PUFA as a practical and effective preventative intervention for mTBI. This work is relevant to clinical practitioners, athletic therapists, athletes, and the general population who wish to support their neurological health through the use of dietary or supplementary n-3 PUFA. Further research in both animal and human models is required to explore potential sex-based differences and to elucidate the specific mechanisms n-3 PUFA modulates in the brain to impart its beneficial neuroprotective effects.

## Author contributions

The authors’ responsibilities were as follows – CACL, DWLM, LMH: designed the research project; CACL, JL: conducted the experimental protocol; CACL: conducted all tissue collection and analysis, and primary writer of the manuscript; CACL, LMH: conducted the statistical analysis; and all authors: contributed edits and revisions to initial manuscript, read and approved the final manuscript.

## Data availability

Data described in the manuscript, code book, and analytic code can be made available on request to the corresponding author, David Ma.

## Funding

Funding support was provided by the Natural Sciences and Engineering Research Council grant to DWLM.

## Conflict of interest

DWLM reports financial support was provided by Natural Sciences and Engineering Research Council of Canada. If there are other authors, they declare that they have no known competing financial interests or personal relationships that could have appeared to influence the work reported in this article.
